# Should We Consider Routine Blood Work for Diplopia and Ptosis in a Primary Care Setting?

**DOI:** 10.7759/cureus.60521

**Published:** 2024-05-17

**Authors:** William L Dietlein, Zachary Dickey, Stephanie Aldret

**Affiliations:** 1 Family Medicine, Edward Via College of Osteopathic Medicine, Monroe, USA

**Keywords:** rare diseases, double vision, ptosis, diplopia, myasthenia gravis, physical medicine and rehabilitation, family medicine, internal medicine, neurology, ophthalmology

## Abstract

Myasthenia gravis (MG) is a rare disorder that most commonly presents with ocular symptoms. Despite the highly sensitive blood work that can be used to diagnose the disease, it is frequently misdiagnosed until the disease becomes systemic. Literature, however, shows that those who begin treatment with acetylcholinesterase inhibitors before systemic presentation have a better prognosis.

We discuss the case of a patient who presented to the clinic with a chief complaint of diplopia that was subsequently referred to ophthalmology. It was not until lab work was done by a subspecialist that the diagnosis of MG was made. The patient quickly responded to an acetylcholinesterase inhibitor and has since had a great prognosis.

Here, we are advocating for the inclusion of routine lab work in the evaluation of patients who present to the primary care setting with diplopia in the absence of red flag symptoms. This approach aids in deciphering the potential involvement of MG in diplopia or ptosis. While such symptoms justify referral to ophthalmology, logistical challenges often hinder a prompt evaluation. Early diagnosis with the incorporation of routine lab work offers the potential to expedite the diagnosis of a rare disease. In doing so, providers can improve prognosis and potentially mitigate additive medical consultations.

## Introduction

Myasthenia gravis (MG) is a disease that is prevalent in 20 of every 100,000 individuals in the United States [1]. Around 85% of patients affected by MG present with extraocular muscle weakness which can manifest as diplopia, ptosis, or both [1]. Extraocular muscle weakness can then further progress to the involvement of other bulbar muscles and limb musculature [1,2]. A thorough history may reveal classical characteristics of MG which will include a patient’s symptoms worsening with exertion and improving with rest [2]. While the true etiology of the disease is highly debated, it is commonly accepted that it involves antibodies against the postsynaptic nicotinic acetylcholine receptor. Due to this advancement in understanding, treatment targeting this mechanism has led to significant reduction in morbidity and mortality through utilization of acetylcholinesterase inhibitors [2]. 

Due to these significant advancements, we are advocating for the use of routine lab work in patients presenting with extraocular symptoms in the absence of red flag symptoms. This will aid in ruling in or out the attribution of symptoms to MG. Due to the high sensitivity of anti-acetylcholine receptor (anti-AChR) or anti-muscle-specific kinase (anti-MuSK) antibodies (the two most common antibodies found in MG) [3], prompt serological testing is paramount in making the diagnosis of MG. Furthermore, serological testing helps differentiate the multiple variations of MG. While anti-AChRs are most common and present in 70%-85% of cases, anti-MuSKs are found in up to 10% diseases and will have a poor if not inverse response to acetylcholinesterase inhibitors [3]. 

While rare, the development of systemic symptoms can include the involvement of the diaphragm and become life-threatening [3]. Furthermore, the disease can have a significant and detrimental impact on a patient’s health in the absence of a swift and accurate diagnosis.

## Case presentation

A 67-year-old Caucasian male presents to the clinic with a chief complaint of double vision over the last few weeks. The patient reports that during upward gaze, he experiences double vision with one object stacked on top of another. He does state that if he covers one eye, the double vision resolves. Past ocular history is significant for macular degeneration and bilateral cataract extraction with intraocular lens placement. The patient was originally evaluated by an ophthalmologist who believed the complaint was related to a partial fourth nerve palsy or the ongoing progression of macular degeneration. The patient's initial workup consisted of unremarkable erythrocyte sedimentation rate (ESR) and C-reactive protein (CRP) levels. The patient was told to patch the eye and follow up with ophthalmology in three weeks. He was seen by his primary care physician (PCP) for a general wellness visit a few weeks later and was still complaining of diplopia. The patient’s physician ordered a magnetic resonance imaging (MRI) of the brain and an ultrasound of the carotid arteries, both of which were unremarkable. On return to ophthalmology, the patient's diplopia was unresolved, so he was sent for evaluation by a neuro-ophthalmologist. Evaluation by neuro-ophthalmology ultimately led to the ordering of rapid plasma regain (RPR) and fluorescent treponemal antibody absorption (FTA-Abs), antinuclear antibodies (ANA), MG titers, and Lyme titers. The results showed an elevated MG titer, and the patient was subsequently diagnosed with MG (Table 1). The patient responded well to pyridostigmine and has had no complaints at follow-up visits.

**Table 1:** Lab results following neuro-ophthalmology evaluation RPR: Rapid plasma regain; FTA-Abs: fluorescent treponemal antibody absorption; ANA: antinuclear antibodies; MG: myasthenia gravis

Lab test	Result
RPR	Nonreactive
FTA-Abs	Nonreactive
ANA	Negative
MG titer	Positive
Lyme titer	Negative

## Discussion

Case discussion

This case demonstrates support for the use of routine lab work to screen for MG. Over the course of several weeks, the patient scheduled multiple visits with both primary care and specialists, undergoing various imaging procedures all before being diagnosed with a simple blood test. While MG is certainly not a disease you will find high on the differential, the utilization of blood work in screening for MG could aid in a more accurate diagnosis and workup. 

In medicine, we are taught to rule out red flags and start from the least invasive to the most invasive diagnostics. This approach emphasizes the importance of a thorough history and physical exam. It was the subspecialist, the third provider to evaluate the patient, who ascertained the important details leading to the diagnosis. The patient endorsed worsening symptoms throughout the day, a classical symptom of MG.

**Figure 1:**
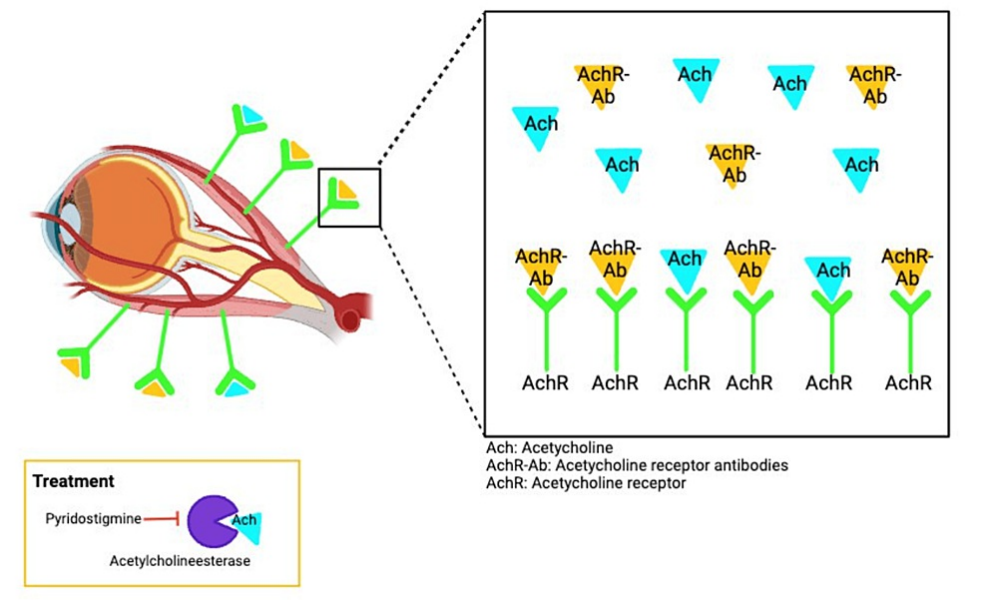
Pathogenesis of ocular myasthenia gravis AchR-Ab: Anti-acetylcholine receptor antibody; ACh: acetylcholine The image shows the pathogenesis of the most common cause of MG with AchR-Ab competing with Ach for Ach receptor binding spots. This image was created by Zachary Dickey via BioRender

While only 10% of all MG cases are purely ocular (Figure 1), 90% of cases that progress to generalized MG occur within the first three years following presentation [4]. Although the incidence of MG is well below 1%, it would be reasonable to consider screening for MG in patients presenting with diplopia and/or ptosis prior to jumping to costly imaging procedures. On initial presentation, blood was drawn to evaluate for giant cell arteritis, a disease with the same incidence (20/100,000) as MG [1,5]. With the check of a box, MG antibody titers similarly could have been run.

Differential diagnosis 

While certain etiologies of diplopia present with obvious ocular motility deficits, oftentimes physical exam findings are more subtle, leaving no certain diagnosis [6]. Extraocular movements involve a network of complex supranuclear circuitry, brainstem nuclei, and cranial nerves [6]. By better understanding structure and function, one is able to construct a more concise differential diagnosis. For example, pure horizontal diplopia suggests medial or lateral rectus dysfunction, while pure vertical diplopia is suggestive of superior oblique dysfunction [6]. This understanding led to the diagnosis of a trochlear nerve palsy in this case. However, this initial diagnosis failed to recognize the significance that MG can play in mimicking palsies as well as other ophthalmoplegia or ocular deviations [6]. This further supports our advocacy of utilizing blood work to rule out MG in similar presentations. Blood work is not limited to MG but can aid in ruling out thyroid myopathies, neurosyphilis, and other atypical etiologies. 

Traditional diplopia workup

**Figure 2:**
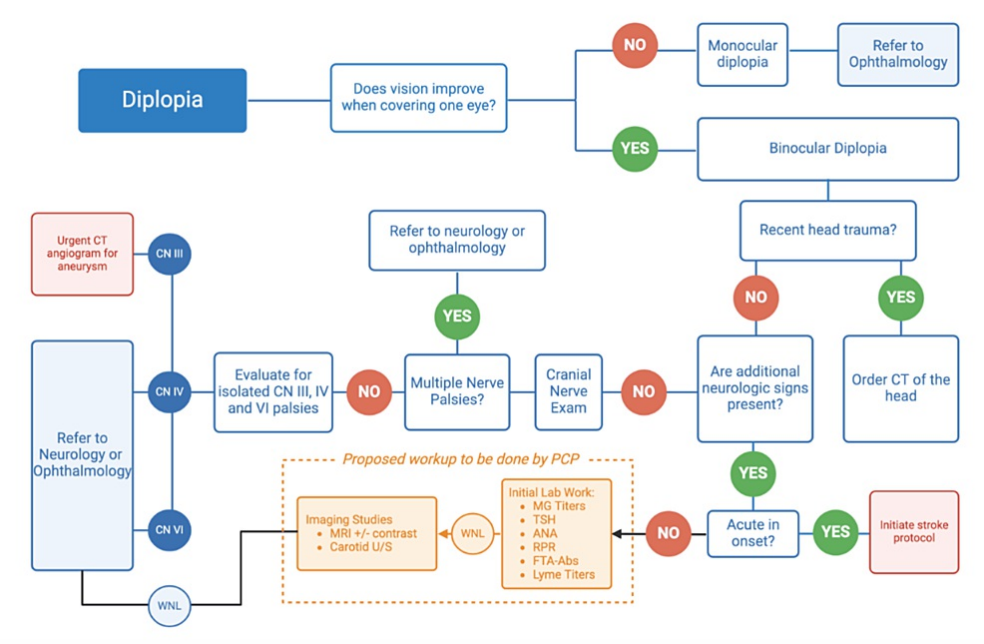
Diplopia workup Diplopia workup including our proposed workup to be completed. This image was created by William Dietlein via BioRender

The first step in a diplopia workup involves classifying the visual disturbance as monocular or binocular diplopia. This is completed by covering one eye. If diplopia persists with one eye covered, it is monocular [7]. If the patient’s symptoms resolve, the diplopia is binocular [7]. Traditional diplopia evaluation from that point is outlined in Figure 2. It is important to note that the majority of the workup has previously been allocated to specialist in ophthalmology and neurology. With the help of routine blood work, we would be able to eliminate the need for specialist referral. This is beneficial, especially for areas in which specialist evaluation is not readily available.

Proposed addition to workup

Although few cases of diplopia will reach an unexplainable point in evaluation, a standard set of labs would aid in catching rare underlying causes. The significance of our case is to advocate for an additional workup to be performed in the primary care setting. We believe this addition will alleviate excessive and unnecessary specialty referrals including neurology and ophthalmology.

Diagnosis and management of MG

Detecting the presence of serum antibodies has become the most universal approach to making the diagnosis of MG. While there are numerous serum antibodies that are sensitive to test for and utilize to make the diagnosis of MG [3,8] , the most common antibody tested for is the anti-acetylcholine receptor antibody (AChR-Ab) (Table 1) [3]. When clinical suspicion is high, however, other serum antibodies are available for testing (Table 1) as well as other pharmacologic and diagnostic tests. For diagnostic purposes, an acetylcholinesterase (AChE) inhibitor that has a short duration of action and immediate onset, such as edrophonium chloride (Tensilon, Enlon), can be used for the diagnosis if patients experience immediate improvement in oculomotor function following injection [8] . Keep in mind that AChE inhibitors may worsen symptoms in MG patients due to muscle-specific kinase antibody (MuSK-Ab) [3]. While not highly sensitive, an ice pack test can be used where ice is placed over the symptomatic eye for 2-5 minutes and improvement in ocular symptoms would be diagnostic [9]. Electromyography (EMG) has the highest negative predictive value [8] and can be completed utilizing the orbicularis oculi muscle [10]; however, finding a provider capable of performing EMG within the ocular region is a major limitation leaving its utilization for systemic or seronegative cases. If completed, however, one would see decreased compound muscle action potentials with repetitive nerve stimulation [8]. A CT scan will be completed following the diagnosis of MG, as a thymoma may be present in up to 15% of patients with AChR-ab [11]. While there remains a variety of tests that can be run to make the diagnosis of MG, it is evident that serum blood work is the most practical place to start. Even more so when a patient presents with symptoms that may not classically align with the diagnosis of MG. 

**Table 2:** Serum antibodies AchR-Ab: anti-acetylcholine receptor antibody; MuSK-Ab: muscle-specific kinase antibody; LRP4-Ab: low-density lipoprotein receptor-related protein 4; Titin-Ab: Titin antibodies; RyR-Ab: ryanodine receptor antibody

Antibody	Prevalence	Clinical association
AchR-Ab	85%	Most common in MG
MuSK-Ab	10%	Poor response to AChE inhibitors
LRP4-Ab	5%	Isolated ocular MG
Titin-Ab	28%	Thymoma
RyR-Ab	23%	Thymoma

## Conclusions

Diplopia can be caused by a variety of etiologies making its workup, from initial history and physical to diagnostic tests, of the utmost importance. Here we advocate for the addition of routine lab work, including MG antibody titers to be performed by a PCP when patients present with binocular diplopia of unclear etiology. While more frequent testing could ultimately result in excessive testing, its utilization can help mitigate healthcare costs and improve the efficiency of diagnosis, in turn improving the prognosis in patients with MG.
